# A Comparative Study of the Effect of Leukoreduction and Pre-storage Leukodepletion on Red Blood Cells during Storage

**DOI:** 10.3389/fmolb.2016.00013

**Published:** 2016-04-21

**Authors:** Thelma A. Pertinhez, Emanuela Casali, Fabio Baroni, Pamela Berni, Roberto Baricchi, Alberto Spisni

**Affiliations:** ^1^Transfusion Medicine Unit, Arcispedale Santa Maria Nuova - IRCCSReggio Emilia, Italy; ^2^Department of Biomedical, Biotechnological and Translational Sciences, University of ParmaParma, Italy; ^3^Department of Surgical Sciences, University of ParmaParma, Italy

**Keywords:** metabolomics, leukoreduction, leukodepletion, NMR, blood transfusion, blood bank conditions

## Abstract

Blood transfusion is a fundamental therapy in numerous pathological conditions. Regrettably, many clinical reports describe adverse transfusion's drawbacks due to red blood cells alterations during storage. Thus, the possibility for a blood bank to ameliorate the quality of the erythrocyte concentrates units is crucial to improve clinical results and reduce transfusion adverse occurrences. Leukodepletion is a pre-storage treatment recognized to better preserve the quality of red blood cells with respect to leukoreduction. Aim of this work is to unravel the biochemical and biophysical basis that sustain the good clinical outcomes associated to the use of leukodepleted erythrocytes units. Erythrocytes concentrates were prepared as leukoreduced (*n* = 8) and pre-storage leukodepleted (*n* = 8) and then studied during 6 weeks in blood bank conditions. Overall, the data indicate that leukodepletion not only provide red blood cells with an appropriate amount of nutrients for a longer time but also selects red blood cells characterized by a more resilient plasma membrane fit to prolong their viability. We believe these results will stimulate new ideas to further optimize the current storage protocols.

## Introduction

In the past years operators in Transfusion Medicine directed particular attention toward the standardization of donor selection, the modality of blood collection, the production of hemocomponents and the development of tests to detect infectious agents. Among the cutting edge themes that are now under discussion (Spitalnik et al., 2015) two of them are particularly relevant: *i*. the identification and quantification of the component of each transfusion product and *ii*. Which criteria must be used to match the available transfusion unit with the patient in specific clinical scenarios. This relevant topic emerges due to the wide clinical variability of transfusion recipients, varying from patients transfused in ambulatory to critical patients requiring multiple transfusions.

Clinical studies identified a correlation between transfusion of red blood cells (RBCs) stored for more than 14 days and the increase of infectious events (Vamvakas and Carven, 1999; Chang et al., 2000; Leal-Noval et al., 2001; Taylor et al., 2002), extension of hospitalization time, prolonged mechanical ventilation, multiple organ failure (Moore et al., 1997) and mortality (Leal-Noval et al., 2003; Gong et al., 2005). The origin of these negative outcomes are attributed to the morphological and biochemical modifications that RBCs undergo during prolonged storage: e.g., the production of lactate leads to a decrease in pH, depletion in adenosine triphosphate and 2,3-diphosphoglycerate. A conclusive solution to those biochemical alterations has not been found yet: storage at low temperature (2–6°C) slows the rate of glycolysis, unfortunately it also decreases the activity of the Na^+^-K^+^ pump, resulting in the alteration of electrolytes balance. The increased K^+^ concentration in the conservation medium augments the risk of arrhythmia in case of rapid transfusion through a central vein. Storage is also known to induce changes in RBCs morphology as well as membrane loss. In this respect, the exact correlation between those storage lesions and the fate of the transfused cells is still unknown and calls the attention of the scientific community (Hess and Grazzini, 2010; D'Alessandro et al., 2012; Dzieciatkowska et al., 2013; Prudent et al., 2015).

A subject of major debate among clinical practitioners is the definition of the criteria for the selection of the transfusion units: the prevalent opinion, at the moment, points to the storage time of the bags. While there are data indicating a correlation between RBCs storage time and morbidity/mortality of the transfused patients (Wang et al., 2012), randomized controlled trials are in progress to verify this fact (Steiner et al., 2010; Lacroix et al., 2011; Alexander et al., 2016).

Consequent to these considerations, reduction of the RBCs storage time would be a simple solution. Unfortunately, it is a practice very difficult to be implemented in a blood bank. Thus, methods to prolong the quality of RBCs during storage are being searched: the removal of leukocytes, before storage, is a promising one.

Beneficial outcomes of leukoreduction and pre-storage leukodepletion, such as the minimization of febrile non-hemolytic transfusion reactions; of cytomegalovirus transmission and anti HLA immunization leading to platelet refractoriness, are well-documented (Eisenfeld et al., 1992; Fischer et al., 1998; Novotny et al., 1995). Other collateral effects, significantly reduced by the introduction of this storage protocol are post-operatory infectious complications, acute respiratory distress syndrome (ARDS), acute lung injury (ALI), transfusion related acute lung injury (TRALI), transfusion-associated circulatory overload (TACO), prolonged mechanical ventilation, hospitalization time and mortality (Bianchi et al., 2015; Silliman et al., 2011).

Recognizing the clinical relevance of these observations, Canada, France and UK adopted universal leukoreduction in 1990, while Germany introduced this pre-treatment in 2001. Until end of 2015, in Italy, RBCs leukodepletion has been used in only few centers and for specific clinical cases. In our hospital, Arcispedale Santa Maria Nuova, Reggio Emilia, Italy, 30% of the RBCs units were leukodepleted and used for critical patients (intensive care unit, cardio surgery, hematology, neonatology). A recent decree of the Italian Ministry of Health, 2 November 2015, designated as mandatory RBCs pre-storage leukodepletion starting January 2016.

The origin of the beneficial outcomes associated to the introduction of RBCs leukodepletion before storage, are expected to be due to the preservation of RBCs quality/vitality for a longer time. Because the main difference between leukoreduced and leukodepleted RBCs is the significantly reduced number of leukocytes present in the bags of leukodepleted RBCs, to progress in the optimization of RBCs storage, it is necessary to understand how the presence of leukocytes influences RBCs vitality, a condition that, at present, is generally associated to the maintenance of both their proper shape and efficient metabolism.

The introduction of new “*omics*” methodologies such as proteomic and metabolomics (Pertinhez et al., 2014; D'Alessandro et al., 2015; Zolla et al., 2015; Nemkov et al., 2016) has provided important tools to answer these questions. In this frame, we have been prompted to study the variation of some RBCs morphological and biochemical parameters during storage. We report here a comparative study on leukoreduced RBCs and pre-storage leukodepleted RBCs.

## Materials and methods

The study was approved by the Arcispedale Santa Maria Nuova (ASMN) Ethics Committee on January 21, 2013. Written informed consent was obtained from all volunteers donors who participated in this study according to the declaration of Helsinki. Blood components were collected from periodical donors of the Transfusion Medicine Unit of ASMN according to the policy of the Italian National Blood Centre Guidelines. Since our experimental conditions did not allow to carried out a paired study, to reduce individual variability we selected 16 males donors aged 30–50 years.

### Blood collection and processing

Sixteen whole blood units (450 mL ± 10%) were collected using the top-and-bottom system (Fresenius Kabi Medicare Bad Homburg, Germany). Eight were collected into triple bags, and eight into quadruple bags, containing Citrate, Phosphate, Dextrose solution (CPD). All the units were centrifuged by Hettich Roto Silenta 630 RS centrifuge (22°C, 11 min, 4000 × g) therefore, most of the plasma and buffy coat was removed using a Compomat G4 separator (Fresenius Kabi Medicare) and RBCs were stored in 100 mL of saline, adenine, glucose and mannitol (SAGM) additive solution. Those collected into triple bags were prepared as Non-Leukodepleted Erythrocyte Concentrate (NLPEC) while the ones collected in the quadruple bags were prepared as Leukodepleted Prestorage Erythrocyte Concentrate (LPEC) using in-line filters.

After 24 h, each RBC unit was divided in 7 satellites bags of 40 mL each (Fresenius Kabi Medicare Bad Homburg, Germany). Satellite bags were stored under standard conditions (2–6°C) and analyzed at different Day (2, 9, 16, 23, 30, 36, and 42) of storage.

An aliquot from each bag was taken for cell count, hematocrit, mean corpuscular value (MCV) and total hemoglobin determination (Sapphire instrument Abbott diagnostic Illinois, USA and Cytomix FC 500 Beckman Coulter IL, Indianapolis, USA). A residual number of leukocytes (< < 1 × 10^6^ a depletion of log 4) were present in the final LPEC units according to the Italian Blood National System regulatory law.

### Definition of NLPEC and LPEC

NLPEC = buffy-coat poor RBCs = leukoreduced RBCs (0.95 ± 0.39 × 10^9^ WBCs per unit, *n* = 8).

LPEC = buffy-coat removal + leukofiltration = highly depleted or leukodepleted RBCs, according to EU guidelines < < 1 × 10^6^ WBCs (a depletion of log 4) per unit.

### RBCs supernatant and RBC lysate preparation

The RBCs supernatant was collected after centrifugation at 2000 × g for 10 min and was divided into two aliquots. One aliquot was used without further modifications for biochemical assays (see below) while the other was depleted of proteins by ultra-filtration (5000 Da cut-off) and frozen at −80°C for subsequent ^1^H-NMR measurements.

RBCs lysate was prepared as follow: red blood cells were washed twice by suspension in 0.9% NaCl in 5 mM phosphate buffer pH 7.4 followed by centrifugation at 2000 g × 10 min. The collected RBCs were then lysed through two cycles of freezing in liquid nitrogen and thawing at 37°C followed by sonication for 30 s. Proteins and membranes were eliminated by ultra-filtration (cut-off 5000 Da), as described in Pertinhez et al. (2014).

### Supernatant biochemical assays

Supernatant: Na^+^ and K^+^ were measured by an indirect ion-selective electrode method (Hoffmann-La Roche Ltd). Total proteins, lactate and lactate dehydrogenase (LDH), were measured by Cobas© Roche.

RBCs hemolysis was evaluated by the absorption spectrum of free hemoglobin (HbO_2_), using an extinction coefficient of 512 mM^−1^cm^−1^ at 415 nm on a spectrophotometer JASCO V-630.

### ^1^H-NMR experiments

Samples were prepared by mixing 570 microliters of the ultra-filtrate either of the RBCs supernatant or of the RBCs lysate, with 30 microliters of TSP (1% in D_2_O) and 10 microliters of 1 M phosphate buffer pH 7.4 (Pertinhez et al., 2014).

1D ^1^H-NMR spectra were acquired at 25°C on a Spectrometer Varian Inova 600 MHz (Palo Alto, USA); processing and peaks assignment was performed with Chenomx NMR suite 7.6 (Edmonton, Canada) as previously described in Pertinhez et al. (2014). The ^1^H-NMR spectra were automatically reduced into consecutive integrated spectral regions (buckets) of an equal width (0.03 ppm). The region containing the water resonance (4.5–5.0 ppm) was not included in the analysis.

### Statistical analysis

Significance has been evaluated by two-way ANOVA tests followed by Fisher LSD *post hoc* tests. *P*-values smaller than 0.05 were considered to be significant.

MestReNova 8.1 software (Santiago de Compostela, Spain) was used to perform the principal component analysis (PCA). Prior to multivariate statistic, pareto scaling, which scales data by dividing each variable by the square root of the standard deviation, was applied.

Note that metabolites concentration were measured starting from the 2^nd^ day of conservation (that therefore is our initial time, t1), after mandatory tests performed by the Transfusion Medicine Unit in accordance with the Transfusion Regulatory Italian law (n° 219, 21 October 2005).

## Results

### RBCs mean corpuscular value during storage

**Figure 1:**
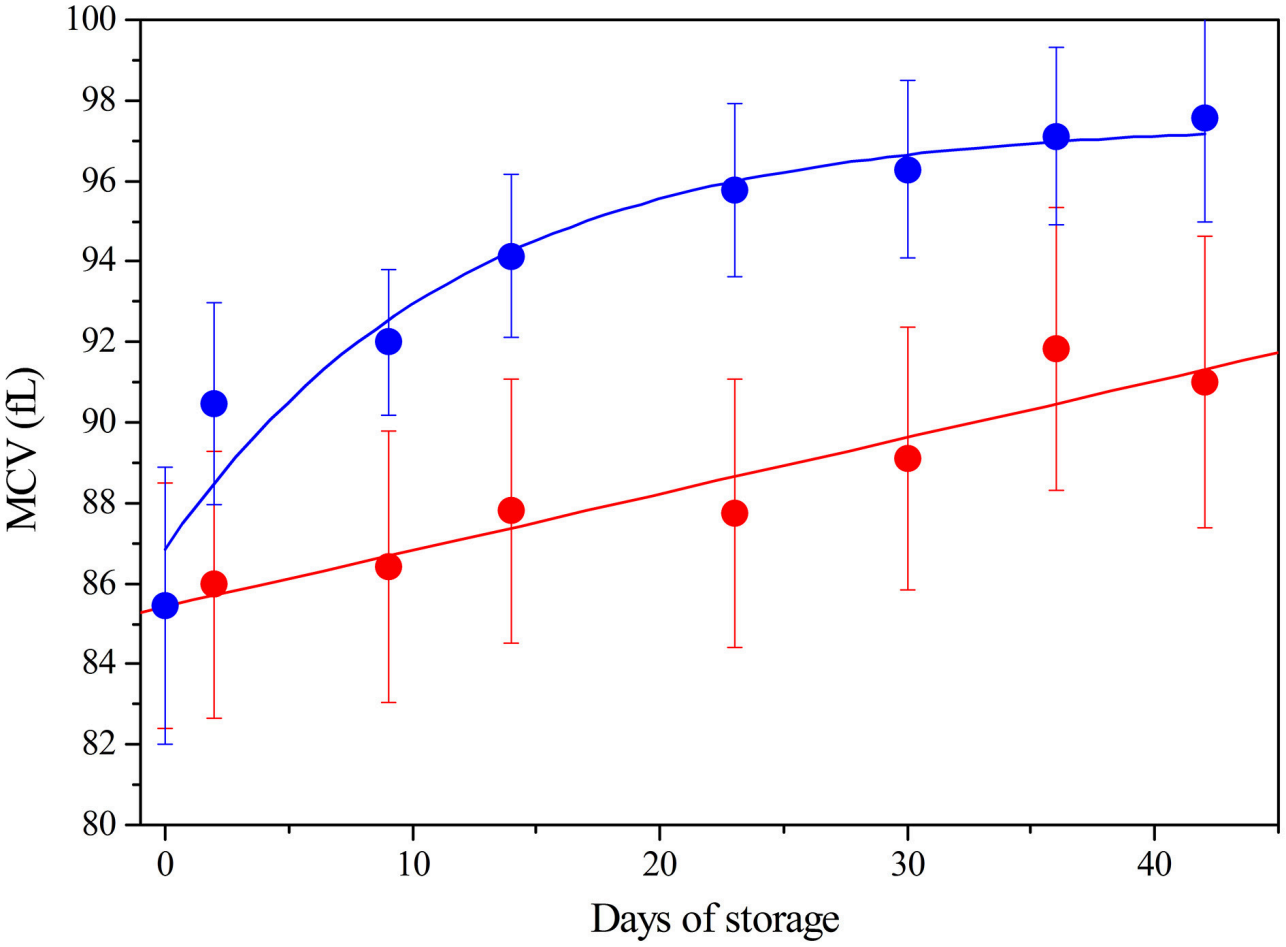
**Mean Corpuscular Value (fL) of RBC during storage: LPEC (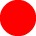) linear fit *r* = 0.96; NLPEC (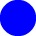) sigmoidal fit *r*^2^ = 0.95 Chi^2^ = 0.137**. ANOVA showed a significant effect for both the storage time and preparation (*p* < 0.0005). Interaction between variables was not significant. *Post hoc* tests indicated always significant differences at each storage time between preparations (*p* < 0.05).

Under physiological conditions, RBCs lifespan is 120 days: their aging *in vivo* is associated to a decrease in volume and an increase in cell density (Bosman, 2013). In blood bank conditions, instead, after the removal of plasma and buffy coat, and re-suspension in SAGM, we observe that RBCs, irrespective of being leukodepleted or not, increase their Mean Corpuscular Value (MCV) during storage (Figure 1).

Note that, in the 16 whole blood freshly collected units, the RBCs showed an initial similar MCV of 85.5 fL ± 2.95 and the MCV values remained within the laboratory reference range (80-100 fL), for both preparations, throughout the storage time.

Nonetheless, Figure 1 shows that NLPEC MCV undergoes a higher increase with respect to LPEC MCV. The increase of red blood cells MCV, which we measure in SAGM, is consistent with the current data (Veale et al., 2011).

The different behavior between NLPEC and LPEC is already evident at time 1, Day 2, (*p* = 0.011) and it becomes more significant at Day 42 (*p* = 0.006). The linear increment (*r* = 0.96) of the MCV values measured for LPEC, as compared to the NLPEC units, that exhibits a sigmoidal behavior (*r*^2^ = 0.95), suggests that leukodepletion selects a homogeneous RBCs population. In particular, we hypothesize that leukodepleted RBCs are characterized by a higher deformability that would favor their passage through the filter and are more resistant to cell volume impairment during storage.

### Changes in free Hb and lactate dehydrogenase (LDH) concentrations

**Figure 2:**
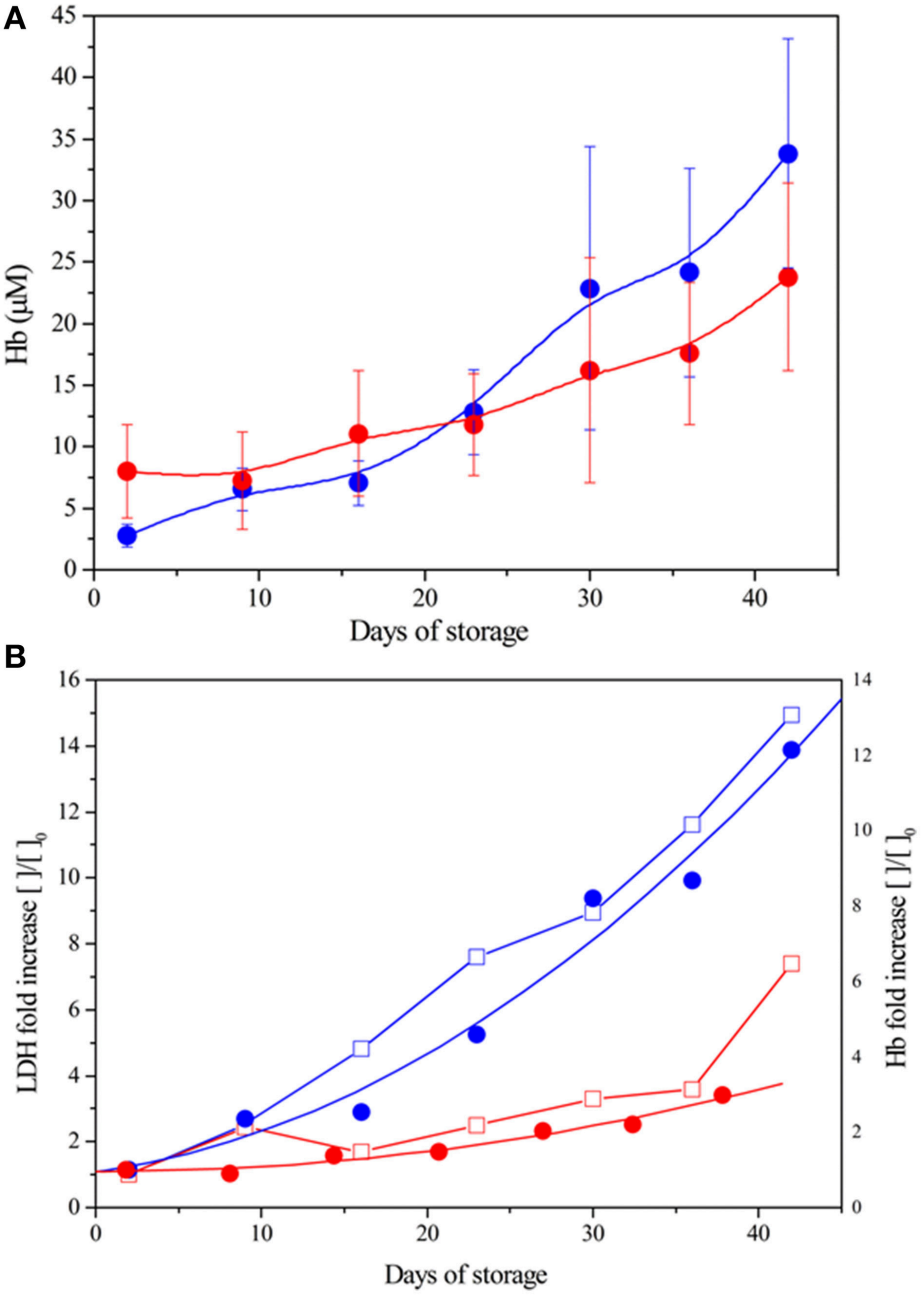
**Hb and LDH in the supernatant of LPEC and NLPEC during storage**. **(A)** Changes in Hb concentration: LPEC (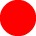), NLPEC (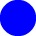). ANOVA showed a significant main effect of storage time (*p* < 0.0005) and significant interaction between storage time and preparation (*p* = 0.0002) **(B)** Fold increase comparison: LPEC LDH (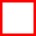) and Hb (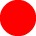), NLPEC LDH (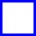) and Hb (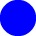).

The free hemoglobin content in the supernatant progressively increases over time for both preparation (Figure 2). This is expected to be the consequence of aging and death of the blood cells with consequent release of the intracellular content in the conservation medium. ANOVA reveals that free hemoglobin concentration is not significantly influenced by preparation (LPEC or NLPEC) but by a combined effect of storage time and preparation. Interestingly, as reported in Figure 2A, in the initial 20 days of storage, NLPEC (Hb = 2.8 μM, Day 2) exhibit a free hemoglobin content that is regularly lower than in LPEC (Hb = 7.9 μM, Day 2); a fact that we attribute to the consequence of leukodepletion, i.e., to the mechanical damage that some blood cells suffer passing through the sieve. However, after Day 20, the trend is reversed and LPEC exhibit a reduced degree of hemolysis, proving that the leukodepleted RBCs are more resistant to the osmotic changes experienced during storage.

To remove any possible bias, data were normalized to the values measured at time 1, Day 2 (see Material and Methods). Figure 2B shows a higher free Hb content in the NLPEC units since the beginning of storage. More than that, in the case of LPEC units, the increment of free hemoglobin is clearly reduced throughout the storage time. The fact that the variation in free Hb is associated to the release of the intracellular enzyme LDH, a marker of cellular damage, assures that we are observing the result of cells' aging and death with subsequent lysis. The correlation between Hb and LDH increase is good in both preparation, LPEC and NLPEC (*r* = 0.93 and 0.97, respectively).

### Electrolytes (K^+^/Na^+^) concentration in the supernantant during storage

**Figure 3:**
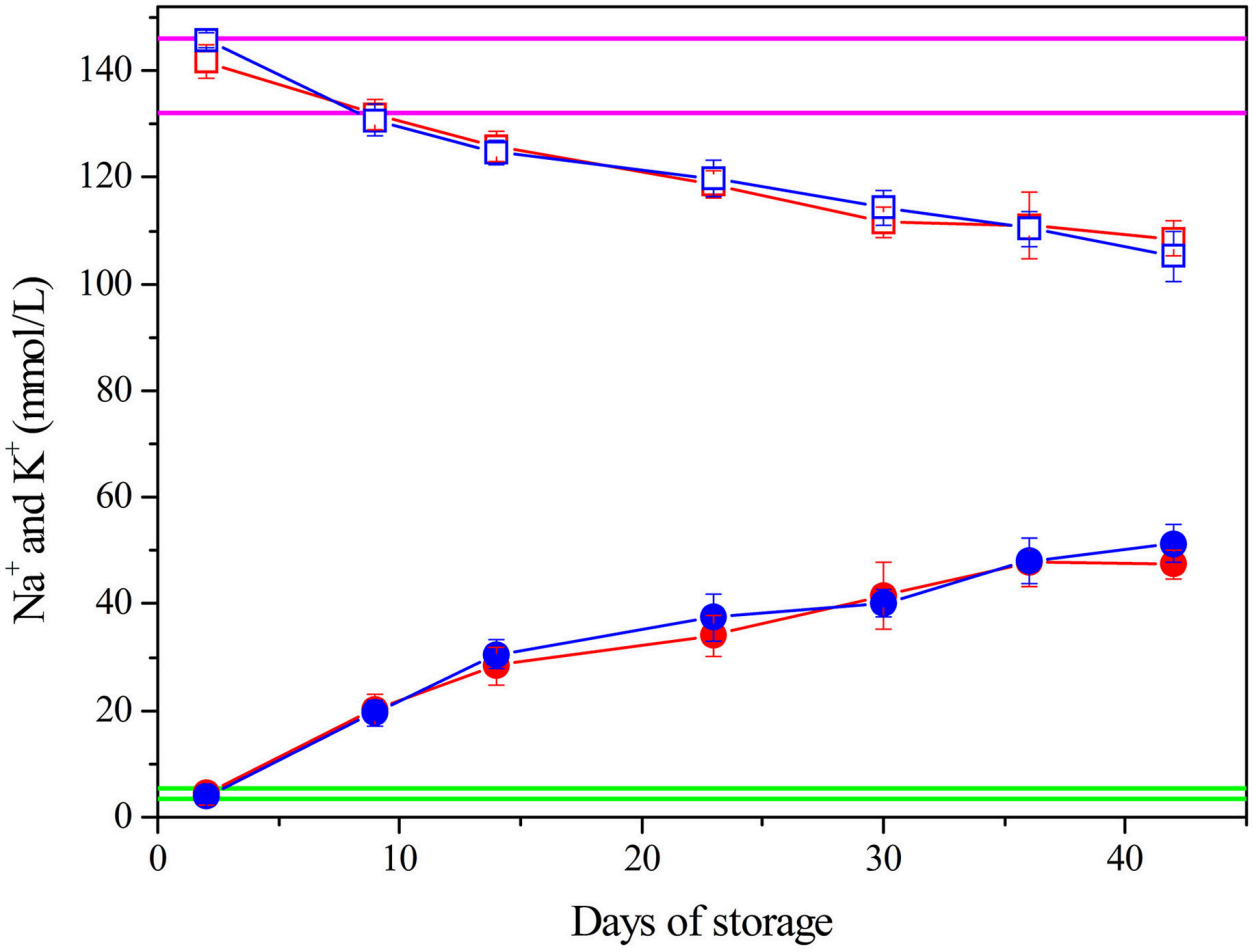
**Electrolytes concentration. K^+^: LPEC (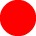), NLPEC (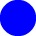). Na^+^: LPEC (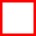) NLPEC (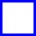)**. Normal ranges are marked in green for K^+^, in magenta for Na^+^. ANOVA showed a significant effect of storage time (*p* < 0.0005) for both ions. Interaction between variables is not significant. *Post hoc*-tests between storage times of the same preparation were always significant (*p* < 0.05) both for LPEC and NLPEC.

Figure 3 shows that for both LPEC and NLPEC the K^+^ and Na^+^ concentration in their supernatant changes across storage period with same trend and extension: K^+^ concentration increases up to ~50 mM at Day 42, while the Na^+^ concentration exhibits a 26% decrease. Our data are consistent with the values reported in the literature and, as generally accepted, result from the low storage temperature.

### Proteins and free amino acids

**Figure 4:**
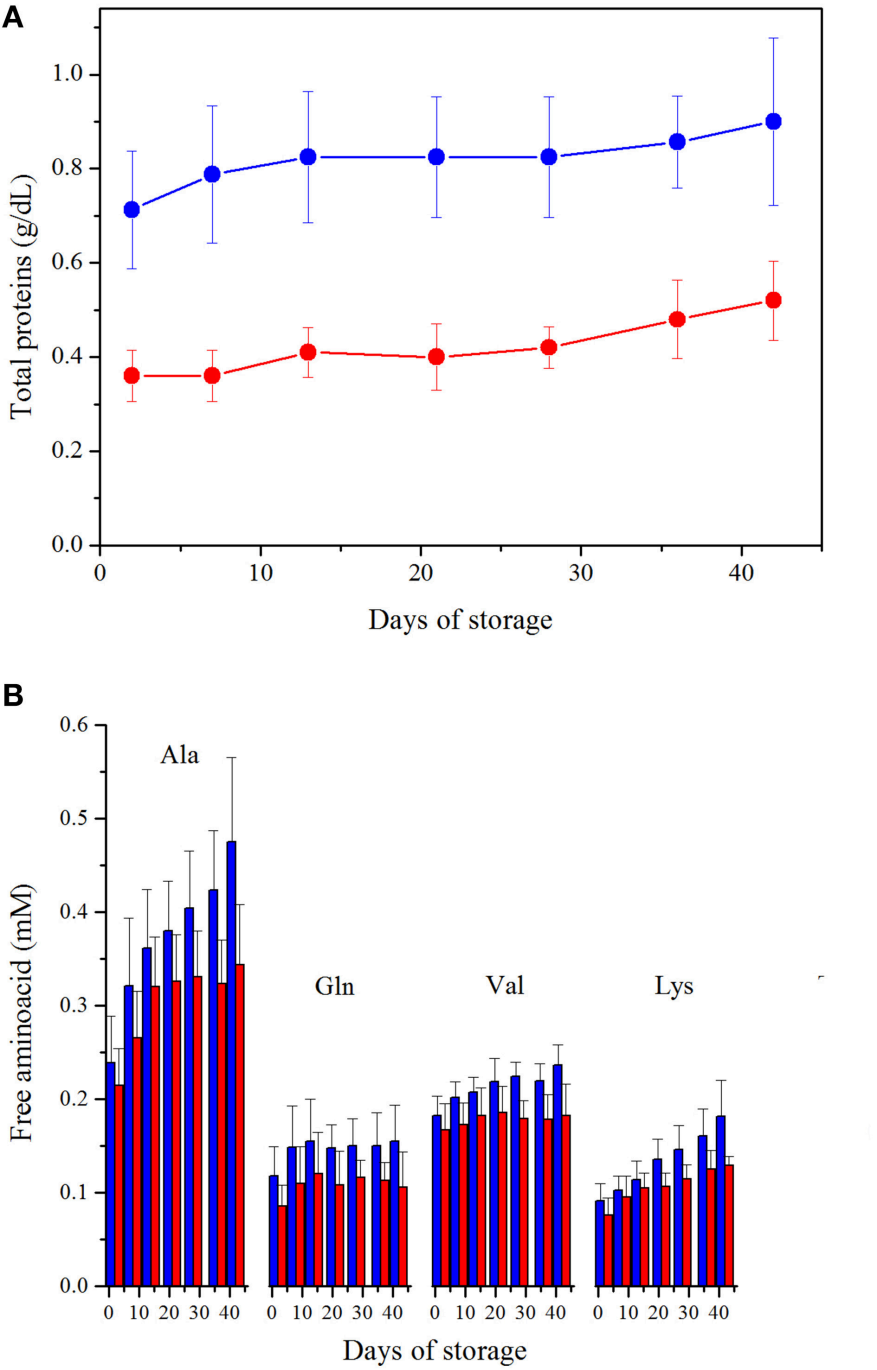
**(A)**. Protein content. ANOVA showed significance (*p* < 0.0005) for both storage time and preparation. Interaction between variables is not significant. LPEC vs. NLPEC comparison at each storage time is always significant (*p* < 0.0001). **(B)** Free amino acids quantification, in the supernatant, during storage. LPEC (red bar) vs. NLPEC (blue bar) comparisons at each storage time addressed all amino acids concentrations significantly higher (*p* < 0.05) in NLPEC units from Day 21.

The supernatant of leukodepleted RBCs exhibits a significantly reduced total proteins content with respect to non-leukodepleted, since Day 2, (*p* < 0.0001), with a Δ-reduction of about 0.4 g/dL maintained over time (Figure 4A). Indeed, in the case of LPEC part of the residual plasma proteins are expected to be filtered out. During storage, an increase of the total protein content is observed in both preparations (≈0.2 g/dL, *p* < 0.001, Day 2 vs. Day 42).

Using ^1^H-NMR spectroscopy, we have been able to identify 11 free amino acids (Gly, Ala, Gln, Phe, His, Tyr, Trp, Leu, Ile, Val, Lys) in the supernatants. Two-way ANOVA indicated a significant effect of both storage time and preparation (*p* < 0.0005) over amino acids concentrations. Ala, Gln, Val and Lys starting from Day 21 (Figure 4B) were always significantly higher in NLPEC (*p* < 0.05). As for the other amino acids, instead, we found a significant higher concentration in NLPEC (*p* < 0.05) starting either from Day 28 or Day 36 (data not shown). These results suggest that in the NLPEC units the extent of cell lysis throughout the storage time is constantly higher than in LPEC. This event leads to a concentration of proteases that is progressively higher in the NLPEC units than in LPEC and, therefore, to an enhanced protein degradation in the NLPEC units: note that during storage WBCs number, in NLPEC, decreases from 0.95 × 10^9^/unit at Day 2 to 0.32 × 10^9^/unit at Day 42.

### Variation of additives concentration in the storage medium

**Figure 5:**
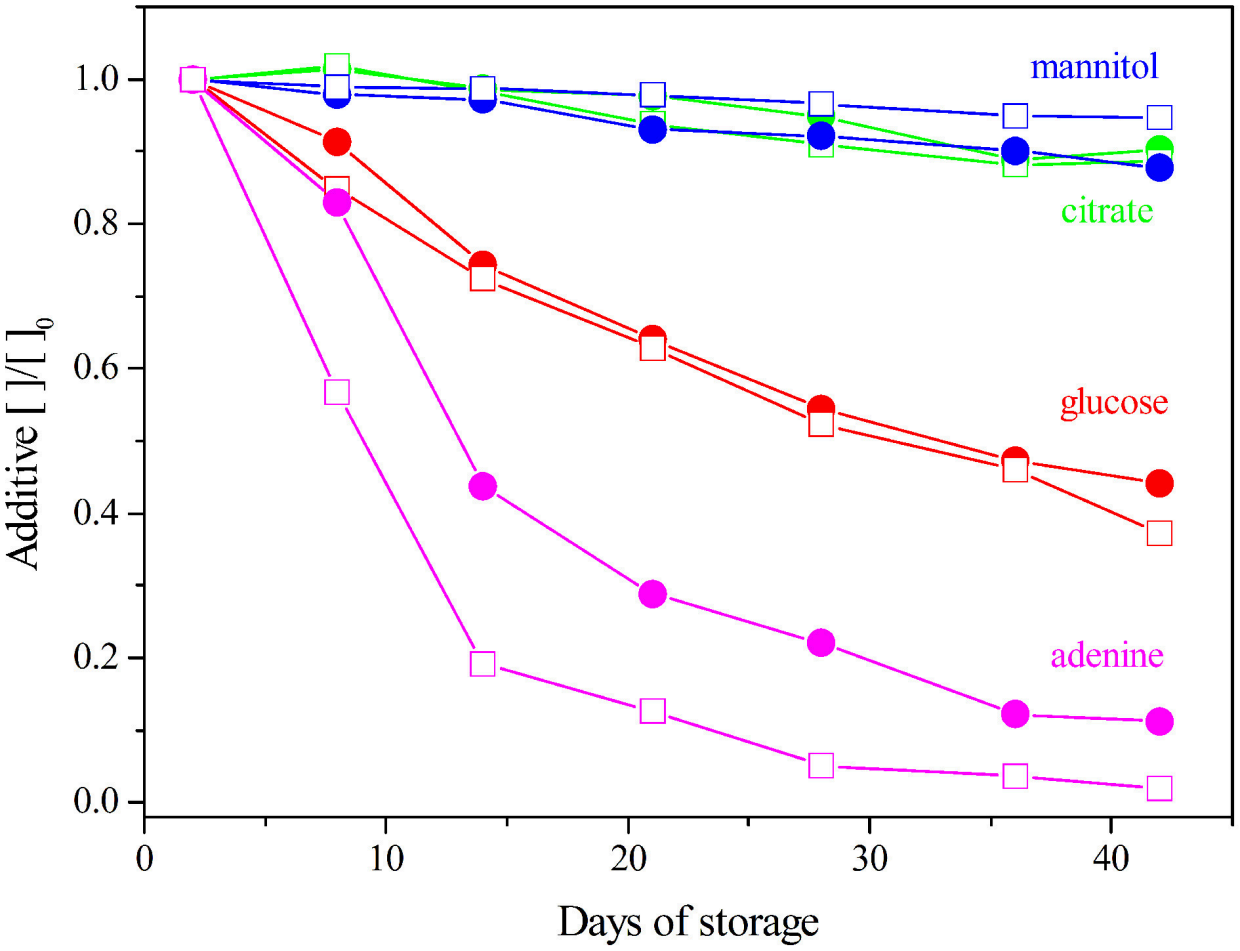
**SAGM additives quantification during storage**. The concentrations are normalized to the Day 2. LPEC (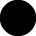) and NLPEC (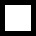).

Figure 5 reports the variation, during storage, of the concentrations of SAGM components measured by ^1^H-NMR. Citrate concentration is stable and comparable, over time, in both NLPEC and LPEC samples as previously reported (Pertinhez et al., 2014). Gevi et al. (2012) reported that in RBCs, stored in SAGM, mannitol decreases overtime. Our results reveal a slight decrease of mannitol concentration in LPEC. Knowing that mannitol is not metabolized, this result points to a facilitated diffusion of this additive inside leukodepleted RBCs.

In NLPEC samples we measure a faster decrease of adenine concentration, this behavior being prominent in the initial 15 days. We interpret this trend as the result of the competition of WBCs and platelets with RBCs for that metabolite that is essential to produce ATP. After Day 15 when apparently most of those cells begins to lyse, as we inferred from the increase of the protein and free amino acid content in the supernatant (see above), the rate of adenine consumption equalizes between the two preparations.

As for glucose consumption, Figure 5 shows it is comparable in both preparations. This result, that at first glance appears contradictory with the trend of adenine depletion, can be rationalized if we recall that in SAGM, the concentrations of glucose (45.4 mM) and adenine (1.25 mM) are quite different, and that the number of WBCs and platelets is considerably smaller than the number of RBCs. Therefore, considering the dynamic range of our measurements, the metabolic activity of WBCs and platelets results more evident when measuring the variation of adenine concentration with respect to the extent of glucose variation.

Overall, these results clearly indicate a negative effect of the presence of WBCs and platelets on RBCs viability during the storage time, as they significantly reduce adenine concentration that turns out to be a limiting factor. In fact, a significant difference is observed through the first 21 days (*p* ≤ 0.005).

### Metabolites concentration in RBC lysates

Table 1S reports a list of the 39 identified metabolites in RBC lysates and their concentrations on Days 2 and 42, for both preparations. A one-way ANOVA was performed to highlight which metabolites were influenced by preparation: 12 compounds presented *p* < 0.005 (Table 1). At Day 2, LPEC present a higher concentration of adenine, citrate, mannitol, and glucose. Alanine and urea, instead, are present at lower concentration. Before any handling, the RBCs cytosolic concentration of those molecules, with respect to SAGM, is lower for adenine, citrate, mannitol and glucose and higher for alanine and urea; we interpret the data obtained for LPEC at Day 2 as the result of filtration. In fact during filtration, it is reasonable to expect that the perturbation of the membrane organization may enhance its osmotic permeability favoring the intake of the SAGM components, and the exit of the cytosolic metabolites. Similarly, we observed significant differences of the cytosolic concentration of some metabolite (*p* < 0.05), at Day 42. The ATP concentration in NLPEC samples is almost 50% lower with respect to LPEC, while purine's degradation catabolites, AMP, IMP and hypoxanthine show increased concentrations (2.5 fold AMP and 1.4 fold IMP and hypoxanthine). In addition, in NLPEC a decrease of glutathione (GSH) is accompanied by an increase of 5-oxoproline, thus pointing to the possibility that, during storage, non-leukodepleted RBCs are more keen to suffer a reduction of their antioxidant defenses, (Pertinhez et al., 2014).

**Table 1:** **Metabolites identified on RBCs lysates of NLPEC and LPEC. Concentration as reported as mean ± SD at Day 2 and Day 42**.

**Metabolite**	**NLPEC (mM)**		**LPEC (mM)**	
	**Day 2**	**Day 42**	**Day 2**	**Day 42**
Adenine^*^	0.119 ± 0.004	0.009 ± 0.006	0.220 ± 0.073	0.017 ± 0.024
Alanine^*^	0.388 ± 0.078	0.426 ± 0.073	0.309 ± 0.031	0.340 ± 0.045
**AMP**	0.140 ± 0.059	0.476 ± 0.160	0.137 ± 0.035	0.188 ± 0.048
**ATP**	1.228 ± 0.184	0.217 ± 0.093	1.425 ± 0.218	0.445 ± 0.089
Citrate^*^	0.017 ± 0.020	0.020 ± 0.011	0.056 ± 0.042	0.036 ± 0.022
Glucose^*^	6.319 ± 1.565	3.235 ± 1.105	11.960 ± 3.897	3.405 ± 1.069
**Glutathione**	2.378 ± 0.426	1.365 ± 0.165	2.692 ± 0.533	1.981 ± 0.529
**Hypoxanthine**	0.015 ± 0.001	0.393 ± 0.131	0.017 ± 0.025	0.280 ± 0.056
**IMP**	0.106 ± 0.038	0.145 ± 0.036	0.082 ± 0.020	0.104 ± 0.029
Mannitol^*^	0.324 ± 0.121	1.421 ± 0.486	0.483 ± 0.019	2.222 ± 0.261
**5-oxoproline**	0.086 ± 0.027	0.710 ± 0.064	0.076 ± 0.031	0.646 ± 0.031
Urea^*^	0.459 ± 0.120	1.005 ± 0.298	0.238 ± 0.113	0.490 ± 0.124

*Metabolites were significantly affected by preparation (ANOVA, p < 0.005). Post hoc tests were performed. In red are highlighted the metabolites whose concentrations at Day 42 are significantly different (p < 0.05) between NLPEC and LPEC*.

* *indicates the metabolites that significantly different (p < 0.05) at Day 2 between both preparations*.

### Chemometric analysis of RBC lysates

**Figure 6:**
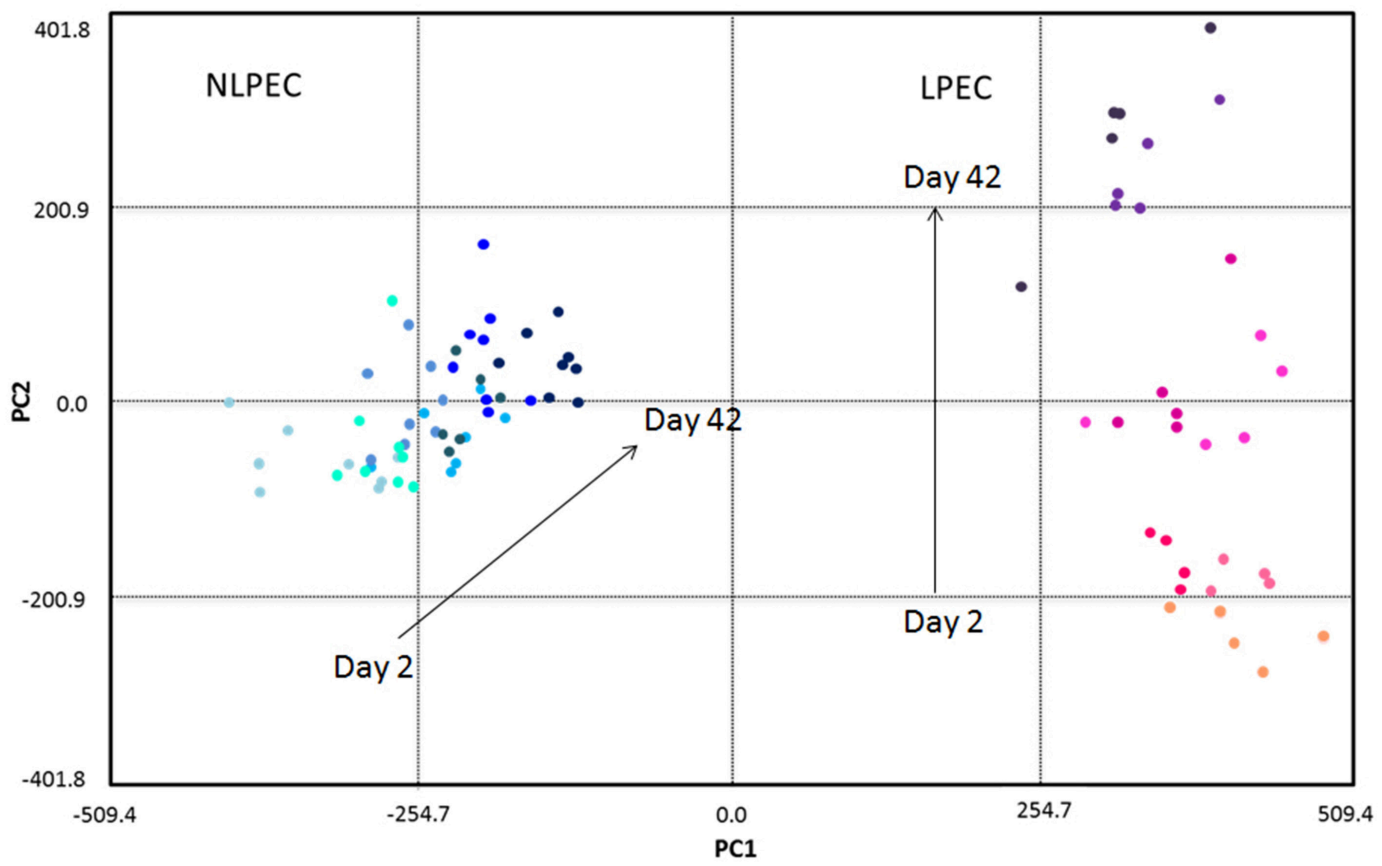
**Chemometric analysis of RBC lysates samples**. The score plot reports NLPEC samples (light to dark blue symbols) and LPEC (orange to violet symbols). PC1 separates on the x axis the two preparations with the major contribution of glucose, lactate and alanine. Mannitol, ATP and GSH contribute to PC2, that on the y axis separate each preparations by day of storage.

As described in previous works (Pertinhez et al., 2014; Casali et al., 2015), using ^1^H-NMR spectroscopy we identified a number of metabolites in the suspension medium of LPEC. However, due to the high concentration of the additives, as well as of the lactate produced by the cells during storage, that covered a wide region of the ^1^H-NMR spectra, the Principal Component Analysis (PCA) of the supernatants composition could not be carried out. The PCA was then performed on LPEC and NLPEC lysates (Figure 6).

The first principal component (PC1) accounts for 63.3% of the total variation in the dataset and well separate LPEC (orange to violet symbols) from NLPEC (light to dark blue symbols). Glucose is the major contributor to PC1 together with alanine and lactate that however exert a minor influence (data not shown).

**Figure 7:**
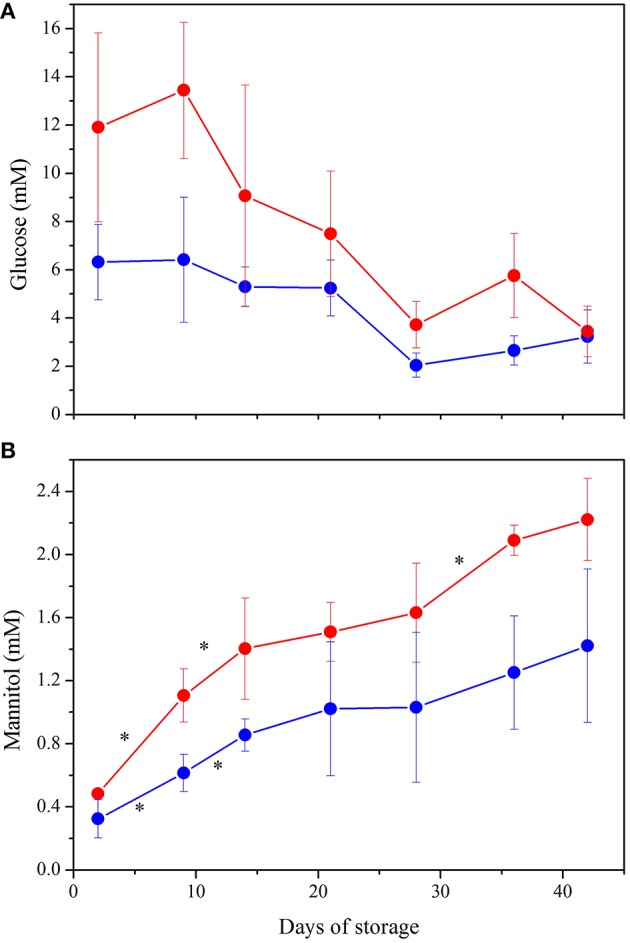
**NMR quantification of the major contributors to PCA plot: (A) Glucose**. ANOVA indicated significant interaction between storage time and preparation (*p* < 0.001) as well as a significant effect of both variables alone (*p* < 0.0005). *Post hoc* tests showed higher concentration of glucose in LPEC from Day 2 to Day 36 (*p* < 0.05). **(B)** Mannitol. Storage time and preparation affect independently mannitol concentration (*p* < 0.0005 for both variables). ^*^indicate *p* < 0.05 when comparing each storage time with the following one, in both preparations. Samples NLPEC (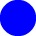), LPEC (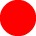).

PC2 accounts for 11.6% of the discrimination among RBCs content and allows to evaluate the changes that occur in the metabolites present in the cytoplasm during the storage. The separation of RBCs as a function of their age is particularly evident for LPEC (Figure 6, right side). Mannitol turns out to be the major contributor to PC2, with ATP and GSH as additional factors. The ability of mannitol to discriminate RBCs during storage is confirmed by t-student's *p*-values obtained for both LPEC and NLPEC, when comparing each storage time with the following one of samples belonging to the same preparation. This is particularly evident between Days 2, 9, and 16 (Figure 7B).

The wide spread produced by PC2 only for LPEC supports the idea that filtration selects RBCs with similar morphological and biochemical features and that exhibit a comparable aging trend.

Figures 7A,B complement the PCA and allow to better understand the metabolic evolution of NLPEC and LPEC over time. Figure 7A shows that in LPEC, glucose concentration is significantly higher (*p* < 0.01) than in NLPEC, throughout storage, except for Day 42. Interestingly however, while in the initial 9 days of storage LPEC presents a concentration of glucose that is twice as much the one present inside NLPEC, from Day 14 throughout Day 36 the difference reduces to about 30% and equalize at Day 42. Overall these data support the hypothesis outlined above that filtration favors an initial burst of glucose uptake. Figure 7B shows that mannitol diffusion inside RBCs proceed more effectively in LPEC than in NLPEC throughout the storage time, consistently with the decrease of its concentration measured in the supernatant (Figure 5). All these data support the hypothesis that leukodepleted RBCs are characterized by a plasma membrane that preserve, for a longer time, its integrity, a fact compatible with their longer viability.

## Discussion

RBCs modifications over time are well-recognized to be one of the factors responsible for the negative outcomes associated to blood transfusion therapy (Sparrow, 2015). Thus, there is a stringent need to improve the protocols used to prepare RBCs concentrates for blood banks. Nowadays, around the world, both leukoreduction and leukodepletion are accepted as pre-storage protocols as they appears to prolong the viability/quality of RBCs units. A recent decree of the Italian Ministry of Health forces all Italian blood transfusion centers to include pre-storage leukodepletion in the preparation of RBCs concentrates. Because the scientific basis of the good clinical results obtained by reducing the number of leukocytes and platelets in the RBCs units to be stored is still matter of debate, and considering that leukodepletion is a more expensive protocol, we have been prompted to seek the biophysical/biochemical motivations that may justify that choice. Answering those questions will allow to devise a rationale improvement of the current storage protocols.

To tackle this issue we chose NMR spectroscopy in combination with standard biochemical (Gallo et al., 2015). Overall we have been able to follow the consumption of the additives necessary to RBCs survival, even in the presence of their high concentration we identified more than 30 metabolites in the supernatant, and 39 metabolites in the lysates RBCs. Interestingly, some of our data were confirmed by MS in the RBCs additive solution AS-3 (D'Alessandro et al., 2015).

Our results highlighted that leukodepletion, differently from leukoreduction: *i*) selects a homogeneous population of RBCs characterized by a healthier and more deformable plasma membrane that assures a prolonged viability. The stress due to filtration destroys the not perfectly fit RBCs, as revealed by the high hemolysis measured at Day 2 (Figure 2) and favors a burst of glucose uptake by the healthy RBCs (Figure 7A) able to pass through the filter. *ii*) by removing WBCs and platelets provides RBCs with an appropriate concentration of nutrients for a longer time (Figure 5). The removal of WBCs and platelets is particularly relevant with respect to the consumption of adenine that in NLPEC turns out to be a limiting factor for cells viability particularly after Day 28 (Figure 5). Indeed, recognizing that to preserve the RBCs energy requirement, the adenine nucleotide pool, under homeostatic conditions, it requires approximately 2 mM ATP, 0.1 mM ADP, and 0.04 mM AMP (Gibson and Harris, 2002), if we estimate the adenine nucleotide pool in NLPEC and LPEC toward the end of the allowed storage time, based on the data reported in Table 1 and Table 1S, we find that LPEC better preserve the adenine nucleotides balance.

The PCA shows also that while the spread of LPEC determined by PC1 is limited, NLPEC are distributed over a wider range of values. Moreover, if we consider the PC2 component we observe it clearly separates LPEC according to their age, while it has practically no effect on NLPEC. This result further confirms the fact that filtration generates a homogeneous population of RBCs evolving uniformly in time.

We feel these results, *per se*, might already justify the advantage of pre-storage leukodepletion over leukoreduction.

In addition we found that the NLPEC suspension medium presents a higher concentration of free amino acids, especially on late storage times (Figure 4B) that, together with the associated higher proteins' content (Figure 4A), suggests the presence of proteases released by dead blood cells. It is worth noting that reduction of proteins and proteins derivatives in the RBCs units, as it occurs in LPEC, may also reduce unexpected immunoreaction in transfused patients.

In summary, our biochemical and biophysical data point to pre-storage leukodepletion as an appropriate and preferable, with respect to leukoreduction, protocol to preserve the quality of the RBCs over time.

We believe this more in depth vision of the behavior of leukoreduced and leukodepleted RBCs, during storage in blood bank conditions, will stimulate new ideas to further optimize storage protocols.

## Author contributions

Study conception and design: TP, EC, RB, and AS; Acquisition of data: TP, EC, FB, and PB; Analysis and interpretation of data: TP, EC, FB, PB, RB, and AS; Drafting of manuscript: TP, EC, FB, PB, RB, and AS; Critical revision: RB and AS; Final approval of the version to be published: TP, EC, FB, PB, RB, AS.

## Funding

This work was supported by Arcispedale Santa Maria Nuova - IRCCS Reggio Emilia, Italy.

### Conflict of interest statement

The authors declare that the research was conducted in the absence of any commercial or financial relationships that could be construed as a potential conflict of interest.

## Abbreviations

CPD: citrate, phosphate, dextrose
LPEC: Leukodepleted-Pre-storage Erythrocyte Concentrate
NLPEC: Non-Leukodepleted-Pre-storage Erythrocyte Concentrate
NMR: Nuclear Magnetic Resonance
RBCs: red blood cells
SAGM: Saline, adenine, glucose, mannitol
WBCs: white blood cells
MCV: mean corpuscular value
PCA: principal component analysis.
