# Supplementary material for: A Comparative Study of the Effect of Leukoreduction and Pre-storage Leukodepletion on Red Blood Cells during Storage
Source: Front Mol Biosci. 2016 Apr 21;3:13. doi: 10.3389/fmolb.2016.00013 (PMC4839302; doi:10.3389/fmolb.2016.00013)
Supplement: Supplementary file 1 [file Table1.DOCX]

**Table 1S:** Metabolites identified on RBCs lysates of NLPEC and LPEC, and their concentration (± SD) at Day 2 and Day 42.

| Metabolite | NLPEC (mM) | | LPEC (mM) | |
| --- | --- | --- | --- | --- |
|  | Day 2 | Day 42 | Day 2 | Day 42 |
| Acetate | 0.100 ± 0.031 | 0.194 ± 0.072 | 0.102 ± 0.035 | 0.134 ± 0.038 |
| **Adenine*** | **0.119 ± 0.004** | **0.009 ± 0.006** | **0.220 ± 0.073** | **0.017± 0.024** |
| ADP | 0.323 ± 0.083 | 0.178 ± 0.065 | 0.315 ± 0.108 | 0.217 ± 0.041 |
| **Alanine*** | **0.388 ± 0.078** | **0.426 ± 0.073** | **0.309 ± 0.031** | **0.340 ± 0.045** |
| **AMP** | **0.140 ± 0.059** | **0.476 ± 0.160** | **0.137 ± 0.035** | **0.188 ± 0.048** |
| Asparagine | 0.156 ± 0.030 | 0.100 ± 0.025 | 0.104 ± 0.039 | 0.121 ± 0.023 |
| Aspartate | 0.414 ± 0.192 | 0.389 ± 0.155 | 0.359± 0.161 | 0.307 ± 0.117 |
| **ATP** | **1.228 ± 0.184** | **0.217 ± 0.093** | **1.425 ± 0.218** | **0.445 ± 0.089** |
| Betaine | 0.172 ± 0.074 | 0.150 ± 0.048 | 0.176 ± 0.195 | 0.117 ± 0.062 |
| Choline | 0.068 ± 0.051 | 0.065 ± 0.050 | 0.037 ± 0.012 | 0.031 ± 0.010 |
| **Citrate*** | **0.017 ± 0.020** | **0.020 ± 0.011** | **0.056 ± 0.042** | **0.036 ± 0.022** |
| Creatine | 0.707 ± 0.130 | 0.644 ± 0.131 | 0.700 ± 0.129 | 0.668 ± 0.120 |
| Creatinine | 0.070 ± 0.009 | 0.064 ± 0.007 | 0.078 ± 0.020 | 0.054 ± 0.029 |
| Formate | 0.066 ± 0.037 | 0.100 ± 0.046 | 0.089 ± 0.034 | 0.072 ± 0.033 |
| Fructose | 0.507 ± 0.385 | 0.510 ± 0.354 | 0.694 ± 0.346 | 0.863 ± 0.243 |
| Fumarate | 0.037± 0.004 | 0.024 ± 0.005 | 0.032 ± 0.013 | 0.018 ± 0.003 |
| **Glucose*** | **6.319 ± 1.565** | **3.235 ± 1.105** | **11.960 ± 3.897** | **3.405 ± 1.069** |
| Glutamate | 0.330 ± 0.078 | 0.094 ± 0.035 | 0.213 ± 0.113 | 0.078 ± 0.024 |
| Glutamine | 0.555 ± 0.098 | 0.147 ± 0.076 | 0.470 ± 0.069 | 0.106 ± 0.029 |
| **Glutathione** | **2.378 ± 0.426** | **1.365 ± 0.165** | **2.692 ± 0.533** | **1.981 ± 0.529** |
| Glycine | 0.535 ± 0.062 | 1.203 ± 0.130 | 0.467 ± 0.113 | 1.194 ± 0.086 |
| Histidine | 0.150 ± 0.018 | 0.140 ± 0.022 | 0.146 ± 0.030 | 0.161 ± 0.025 |
| Homocysteine | 0.139 ± 0.032 | 0.049 ± 0.015 | 0.287 ± 0.411 | 0.050 ± 0.020 |
| **Hypoxanthine** | **0.015 ± 0.001** | **0.393 ± 0.131** | **0.017 ± 0.025** | **0.280 ± 0.056** |
| **IMP** | **0.106 ± 0.038** | **0.145 ± 0.036** | **0.082 ± 0.020** | **0.104 ± 0.029** |
| Isoleucine | 0.027 ± 0.007 | 0.049 ± 0.007 | 0.031 ± 0.008 | 0.041± 0.008 |
| Lactate | 3.830 ± 0.774 | 26.46 ± 5.231 | 3.038 ± 1.163 | 22.912 ± 3.026 |
| Leucine | 0.062 ± 0.011 | 0.111 ± 0.009 | 0.045 ± 0.014 | 0.071 ± 0.014 |
| Lysine | 0.125 ± 0.035 | 0.116 ± 0.041 | 0.111 ± 0.015 | 0.130 ± 0.031 |
| Malonate | 0.088 ± 0.015 | 0.080 ± 0.015 | 0.077 ± 0.013 | 0.071 ± 0.013 |
| **Mannitol*** | **0.324 ± 0.121** | **1.421 ± 0.486** | **0.483 ± 0.019** | **2.222 ± 0.261** |
| Nicotinurate | 0.110 ± 0.010 | 0.109 ± 0.014 | 0.055 ± 0.008 | 0.059 ± 0.019 |
| **5-oxoproline** | **0.086 ± 0.027** | **0.710 ± 0.064** | **0.076 ± 0.031** | **0.646 ± 0.031** |
| Phenylalanine | 0.051 ± 0.009 | 0.097 ± 0.013 | 0.038 ± 0.013 | 0.072 ± 0.008 |
| Pyruvate | 0.312 ± 0.076 | 0.402 ± 0.168 | 0.200 ± 0.084 | 0.436 ± 0.050 |
| Taurine | 0.183 ± 0.053 | 0.126 ± 0.037 | 0.190 ± 0.078 | 0.150 ± 0.080 |
| Tyrosine | 0.098 ± 0.022 | 0.128 ± 0.022 | 0.096 ± 0.013 | 0.113 ± 0.009 |
| **Urea*** | **0.459 ± 0.120** | **1.005 ± 0.298** | **0.238 ± 0.113** | **0.490 ± 0.124** |
| Valine | 0.100 ± 0.016 | 0.161 ± 0.014 | 0.066 ± 0.015 | 0.112 ± 0.009 |

In red are highlighted the metabolites whose concentrations at Day 42 are significantly different (p<0.05) between NLPEC and LPEC. * indicates the metabolites that significantly different (p<0.05) at Day 2 between both preparations.
